# Meniscal Allograft Transplantation Failure Is Associated With Female Sex: A 15-Year Survival Analysis

**DOI:** 10.1177/03635465261470172

**Published:** 2026-08-11

**Authors:** Yunseo Linda Park, Anja M. Wackerle, Charles Jiang, Romed P. Vieider, Philipp W. Winkler, James J. Irrgang, Volker Musahl, Jonathan D. Hughes

**Affiliations:** * University of Pittsburgh School of Medicine, Pittsburgh, Pennsylvania, USA; † Department of Orthopaedic Surgery, UPMC Freddie Fu Sports Medicine Center, Pittsburgh, Pennsylvania, USA; ‡ Department for Orthopaedic Sports Medicine, Klinikum Rechts der Isar, Technical University of Munich, Munich, Germany; § Department of Physical Therapy, University of Pittsburgh, Pittsburgh, Pennsylvania, USA; ‖ Department for Orthopaedics and Traumatology, Kepler University Hospital GmbH, Johannes Kepler University Linz, Linz, Austria; Investigation performed at UPMC, Pittsburgh, Pennsylvania, USA

**Keywords:** meniscal allograft transplantation, graft survival, patient-reported outcomes, risk factors, biological knee reconstruction, gender

## Abstract

**Background:**

While short- and midterm outcomes after meniscal allograft transplantation (MAT) are well documented, there is a paucity of data regarding long-term graft survival.

**Purpose:**

To assess mid- to long-term graft survivorship and patient-reported outcomes (PROs) after MAT.

**Study Design:**

Cohort study; Level of evidence, 3.

**Methods:**

All patients who underwent MAT with bone block or soft tissue fixation between 1993 and 2020 at a single institution were identified. Individuals with <5 years of follow-up and untreated grade 4 cartilage lesions were excluded. Primary outcomes included graft survivorship and long-term PROs including International Knee Documentation Committee Subjective Knee Form (IKDC SKF) score, Knee injury and Osteoarthritis Outcome Score, and Marx Activity Scale score. Graft failure was defined as the need for meniscal allograft resection or repair, revision MAT, or conversion to total knee arthroplasty. Clinical failure was defined as an IKDC SKF score <56 at the final follow-up. Statistical analyses included chi-square/Fisher exact tests for categorical variables, the Mann Whitney *U* or *t* test for continuous variables, and Kaplan-Meier survival analysis with endpoints as graft failure. Statistical significance was defined as a *P* value <.05.

**Results:**

This study included 160 patients (57% of the screened population; 46% female; median age at surgery, 28 years) with a median follow-up of 15 years (IQR, 10-21 years). A total of 43 graft failures occurred during follow-up, representing an 84% survival rate at 10 years, 76% at 15 years, 69% at 20 years, and 52% at 25 years. On univariable Cox regression, no patient or surgical factors, including age, sex, body mass index, tobacco use, fixation method, cartilage status, or concomitant osteotomy, were significantly associated with both graft and clinical failure (all *P* > .05). However, after adjusting for age and cartilage grade in the MAT compartment, female sex became a significant predictor for graft failure (*P* = .04). Clinical failures occurred in 32 patients (40%) and were not associated with graft failures (*P* = .20). PROs were not significantly different between male and female patients (*P* > .05).

**Conclusion:**

The MAT survival rate was 84% at 10 years, with a gradual decline to 76% at 15 years, 69% at 20 years, and 52% at 25 years. After adjusting for age and presence of cartilage lesion, female sex emerged as a predictor of graft failure. These findings highlight the importance of careful patient selection and counseling regarding long-term expectations after MAT.

Meniscal allograft transplantation (MAT) is a joint-preserving surgical option for young, active individuals with postmeniscectomy knee pain. Outcomes after MAT have shown sustained improvement in pain and function,^11,30^ with a meta-analysis in 2022 demonstrating postoperative patient-reported outcome (PRO) scores exceeding minimal clinically important difference (MCID) thresholds at follow-up lengths ranging from 1 to 12 years.^29^ Individuals also reported high satisfaction; in a recent retrospective review of 174 persons who underwent MAT with a minimum 10-year follow-up, 86% indicated they were satisfied with their overall postoperative outcome.^8,35^

Despite these benefits, MAT carries risks of complications. Rates of reoperation to the index meniscus after MAT have been reported to be as high as 37% by 10 to 15 years, while rates of revision MAT or conversion to total knee arthroplasty (TKA) range between 7% and 15%.^4,35^ Additionally, while short- and midterm outcomes are encouraging, there are fewer studies providing robust long-term data beyond 10 years. Existing long-term studies are often limited by loss to follow-up and incomplete data, which may bias survival estimates and hinder evaluation of late failures.^17,23,30,33-35^

The current literature on failure after MAT lacks standardized definitions of failure. Criteria for graft survival are heterogeneous and include clinical failure (eg, Lysholm score <65), anatomic failure (eg, meniscal tear or extrusion), and surgical failure (eg, graft removal or conversion to TKA). Although some studies identify factors such as age, sex, body mass index (BMI), cartilage lesions, and concomitant procedures as predictors of failure,^10,12,15^ the magnitude and interaction of these risks are not fully elucidated.

The purpose of this study was to assess mid- to long-term survivorship, defined as the absence of graft resection, repair or revision, or conversion to TKA, as well as evaluate risk factors for failure and PROs after MAT. We hypothesized that MAT would demonstrate mid- to long-term survivorship similar to the current literature, and factors such as older age and presence of cartilage lesions would be associated with an increased risk of graft failure. We also hypothesized that PROs after MAT would be maintained at high levels at long-term follow-up.

## Methods

This study was approved by the institutional review board (STUDY22110002). This study was a retrospective review. Written informed consent was acquired on inclusion of the participant into the study. All individuals who underwent primary MAT between 1993 and 2020 at the authors’ institution by 4 surgeons were screened for eligibility. Study participants were included if they underwent primary medial or lateral MAT, had bone block or soft tissue fixation methods, were ≥14 years of age at the time of surgery, had an accessible operative note, and had a minimum follow-up of 5 years. Individuals were included regardless of the presence of concomitant procedures at the time of MAT or before MAT. Follow-up length was determined by the last time the individual was seen by an orthopaedic surgeon or the date that the questionnaire was completed. Patients with untreated International Cartilage Regeneration & Joint Preservation Society (ICRS) grade 4 cartilage lesions at the time of MAT were excluded, while those with grade 4 lesions that were managed at the time of or after their MAT were included.^5^ Data collection was performed via review of the medical records and supplemented by patient-completed questionnaires on descriptive characteristics, sports participation, subsequent operative procedures after MAT, and the following PROs: International Knee Documentation Committee Subjective Knee Form (IKDC SKF) score,^13^ Knee injury and Osteoarthritis Outcome Score (KOOS),^24^ and Marx Activity Scale score.^20^ Concomitant surgeries performed at the time of MAT, as well as those conducted before and after MAT, were recorded. Whether surgeries were staged or preplanned in conjunction with MAT was denoted.

### Surgical Indication and Technique

Individuals were indicated for MAT if they had subtotal or complete meniscal loss, as well as pain and functional limitations. Eligibility for MAT was determined based on history, physical examination, imaging, and arthroscopic evaluation. Limb alignment and ligament stability were assessed before MAT to determine the need for concomitant procedures such as high tibial or distal femoral osteotomy and/or ligament reconstructions. Indication for osteotomy was generally based on surgeon assessment of clinically and radiographically evident malalignment, with the goal of correcting compartmental overload. High tibial osteotomies were performed in patients with malalignment causing increased weightbearing through the ipsilateral compartment. Grade 3 and 4 cartilage lesions were treated via microfracture or osteochondral allograft transplantation. This decision was made at the discretion of the treating surgeon based on lesion size, containment, subchondral bone involvement, and patient-specific factors.

Meniscal allograft sizing was determined preoperatively using standardized measurements on magnetic resonance imaging scans and true lateral and anteroposterior radiographs. Arthroscopically assisted MAT was performed through posteromedial and posterolateral incisions for medial and lateral MATs, respectively, to insert and secure the graft. Meniscal allograft root fixation was achieved via transtibial tunnels with either a suture-only or a bone block technique. For medial MATs, bone plugs from the graft roots were seated into separate tibial tunnels and fixed. For lateral MATs, the anterior and posterior bone blocks were connected by a bone bridge, which was placed into a tibial trough and secured. Bone block fixation was the preferred technique for lateral MAT up until 2010 at our institution, while technique choice for medial MAT depended on surgeon preference, prior or concomitant procedures, and the presence of tibial bone loss. Starting in 2011, the suture-only technique was more commonly performed for both medial and lateral MATs. Additional fixation of the graft body to the native meniscal rim and capsule was achieved with all-inside, inside-out, or outside-in sutures. Postoperatively, all patients followed a standardized rehabilitation protocol, as described previously.^7,21^

### Definition of Failure

Both graft failure and clinical failure were assessed. Graft failure was defined as the need for subtotal or total meniscal allograft resection, meniscal allograft repair, revision MAT, or conversion to unicompartmental knee arthroplasty or TKA.^38^ Arthroscopic debridement or resection of less than one-third of the allograft width was not considered a graft failure. Meniscal allograft repair was included in the definition of graft failure, as it represents that a subsequent surgical intervention was necessary to address the structural compromise of the graft. Time to failure was measured from the date of MAT to the subsequent operation that met these criteria.

Clinical failure was defined as an IKDC SKF score <56 at the final follow-up based on the previously established Patient Acceptable Symptom State (PASS) threshold after MAT.^36^ Unlike graft failure, time to clinical failure could not be determined, as the exact point when a patient's IKDC SKF score fell below 56 is unknown. The recorded date simply reflected when the IKDC SKF score was collected, not when the clinical failure occurred. Return to sport was defined as the individual's report of returning to their primary sport at any level.

### Statistical Analysis

All statistical analyses were conducted using SPSS software Version 28.0.1.1 (IBM). Normality of continuous variables was assessed using the Shapiro-Wilk test in conjunction with visual inspection of data distributions. Parametric variables are reported as mean ± standard deviation, while nonparametric variables are reported as median and interquartile range. Categorical variables are reported as count and percentage of the entire cohort. A survival analysis was performed via the Kaplan-Meier method with graft failure as the endpoint; patients without graft failure were censored at their most recent clinical follow-up. Kaplan-Meier survival curves were used for whole group survival as well as significant predictors of graft failure, as determined by the Cox proportional hazards model. Differences in Kaplan-Meier survival patterns were assessed using the log-rank test. A univariable Cox proportional hazards model was used to determine the effect of each potential predictor variable on graft failure, independently of other factors. A multivariable Cox proportional hazards model was then used with variables of interest and those that approached significance as a potential independent predictor for graft failure. As explained above, clinical failure was a binary outcome with no time variable; thus, a logistic regression was performed to identify risk factors for clinical failure.

To contextualize patient-reported knee function relative to the general population, IKDC SKF scores were also standardized using age- and sex-matched normative data.^1^ For each participant, a standardized IKDC SKF score was calculated as previously described.^1^ Standardized scores reflect deviation from population norms, with a score of 0 indicating knee function equivalent to age- and sex-matched normative values, positive values indicating worse function, and negative values indicating better function relative to the normative population. As a secondary analysis, PROs were compared between male and female patients to assess potential differences in long-term functional outcomes. To compare outcomes between groups, Mann-Whitney *U* tests were used for variables that were not normal, and independent-samples *t* tests were used for normally distributed variables. The level of significance was set at a *P* value <.05.

A priori power analysis was performed to determine the minimum sample size needed to detect a clinically meaningful difference in postoperative PROs between groups. A prior study determined that the MCID for the IKDC SKF score is approximately 11.5 points.^14^ The standard deviation was estimated at 15 based on similar patient populations,^31^ yielding a Cohen *d* of 0.77. With an alpha of .05 and 80% power, a minimum of 29 patients per group was required. The available sample size met this requirement, allowing meaningful comparison of outcomes between men and women with surviving grafts.

## Results

**Figure 1.**
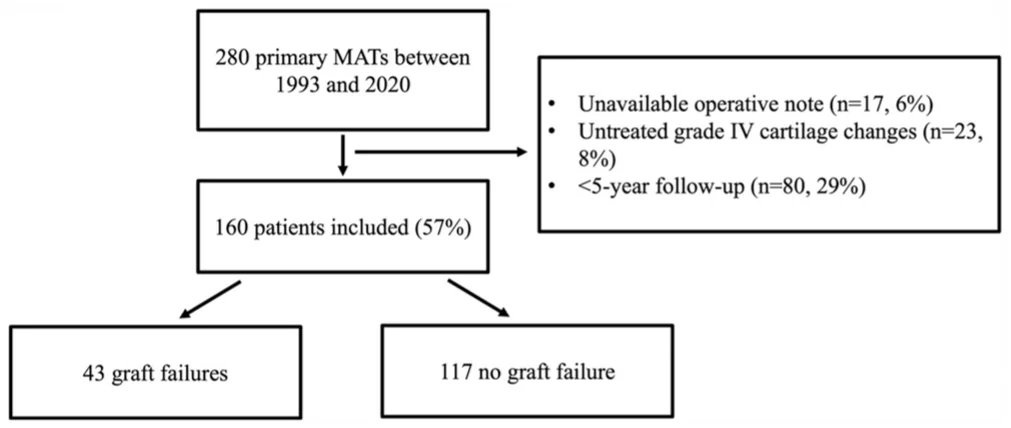
Patient flowchart. MAT, meniscal allograft transplantation.

A total of 280 patients were identified for initial evaluation (Figure 1); after inclusion and exclusion criteria were applied, the study cohort consisted of 160 patients (57% of the screened population) with a median age of 28 years (IQR, 21-36 years) at the time of MAT and a median follow-up of 15 years (IQR, 10-21 years). Two patients had simultaneous bicompartmental MAT and were excluded from all medial versus lateral MAT comparisons. One patient had MAT in both knees and was treated as 2 separate data points. One patient had bicompartmental (both medial and lateral) MAT done at separate time points and was also treated as 2 separate data points.

Baseline characteristics at the time of MAT are shown in Table 1. A detailed summary of operative procedures before and after MAT, both staged and nonstaged, as well as concomitant procedures performed with MAT are listed in Table 2. During follow-up, 93 patients (58% of the study group) underwent 165 subsequent operative procedures. Thus, a mean of 1.8 ± 1.1 (range, 1-6) subsequent operative procedures was observed for patients undergoing reoperation.

**Table 1:** Baseline Characteristics at Time of MAT (n = 160)*^*a*^*

	Value
Descriptive characteristics	
Age at time of MAT, y	28 (21-36)
Female sex	74 (46)
BMI at surgery, kg/m^2^ (n = 124)	27 (24-32)
Follow-up length, y	15 (10-21)
Smoking at time of MAT (n = 141)	
Never	96 (68)
Current	22 (16)
Former	23 (16)
Surgical characteristics	
Right knee	92 (58)
Medial MAT	102 (64)
Bone block fixation technique	57 (36)
Concomitant surgery performed	66 (41)
ICRS grade ≥3 present in any compartment	77 (48)
ICRS grade ≥3 present in MAT compartment	69 (43)
No. of previous operative procedures	2 (1-3)
Time from first operative procedure of index knee, y (n = 119)	5 (2-10)

a Data are presented as median (IQR) or n (%). BMI, body mass index; ICRS, International Cartilage Regeneration & Joint Preservation Society; MAT, meniscal allograft transplantation.

**Table 2:** Detailed Surgical History of Index Knee, Stratified by Chronological Order of Surgeries*^*a*^*

	Value
Nonstaged surgeries before MAT	
Index meniscus	
Partial meniscectomy	140 (88)
Meniscal repair	26 (16)
Opposite meniscus	22 (14)
Nonmeniscus	
Diagnostic arthroscopy/debridement	13 (8)
ACLR	84 (53)
Cartilage surgery	7 (4)
Osteotomy	4 (3)
Other*^*b*^*	6 (4)
Staged (planned) surgeries before MAT	
Any staged surgery	66 (41)
Diagnostic arthroscopy/debridement	61 (38)
Osteotomy	2 (1)
Cartilage surgery	2 (1)
Partial meniscectomy	6 (4)
Ligament surgery	1 (<1)
Other*^*c*^*	14 (9)
Concomitant surgeries at the time of MAT	
Any concomitant surgery	66 (41)
Primary ACLR	24 (15)
Revision ACLR	30 (19)
Other*^*d*^*	12 (8)
Opposite compartment meniscal surgery	8 (5)
Osteotomy	4 (3)
Staged (planned) surgeries after MAT	
PCL	1 (<1)
Nonstaged surgeries after MAT	
Diagnostic arthroscopy/debridement	47 (29)
Total knee arthroplasty	26 (16)
Ligament	
ACLR	11 (7)
MCL repair	3 (2)
Any reoperation on index meniscus	44 (28)
Minor debridement	5 (3)
Meniscal allograft partial meniscectomy	20 (13)
Meniscal allograft subtotal/total meniscectomy	9 (6)
Meniscal allograft repair	8 (5)
Revision MAT	4 (3)
Opposite meniscus	14 (9)
Cartilage surgery	13 (8)
Osteotomy	1 (<1)

a Data are presented as n (%). Staged surgeries refer to those that were planned and performed shortly before or after meniscal allograft transplantation (MAT) as part of a coordinated treatment plan. Nonstaged surgeries refer to those that were not planned in conjunction with MAT, occurring independently either before or after the transplant. ACLR, anterior cruciate ligament reconstruction; PCL, posterior cruciate ligament.

b Hardware removal (n = 2), cyst removal (n = 2), synovectomy (n = 1), bone grafting (n = 1).

c Bone grafting (n = 8), hardware removal (n = 9).

d Hardware removal (n = 4), microfracture or chondroplasty (n = 3), tibial tubercle osteotomy (n = 1), osteochondral allograft transplantation (n = 1), medial collateral ligament (MCL) repair (n = 1), lateral extra-articular tenodesis (n = 1), synovectomy (n = 1).

### Graft Survival Analysis

**Figure 2.**
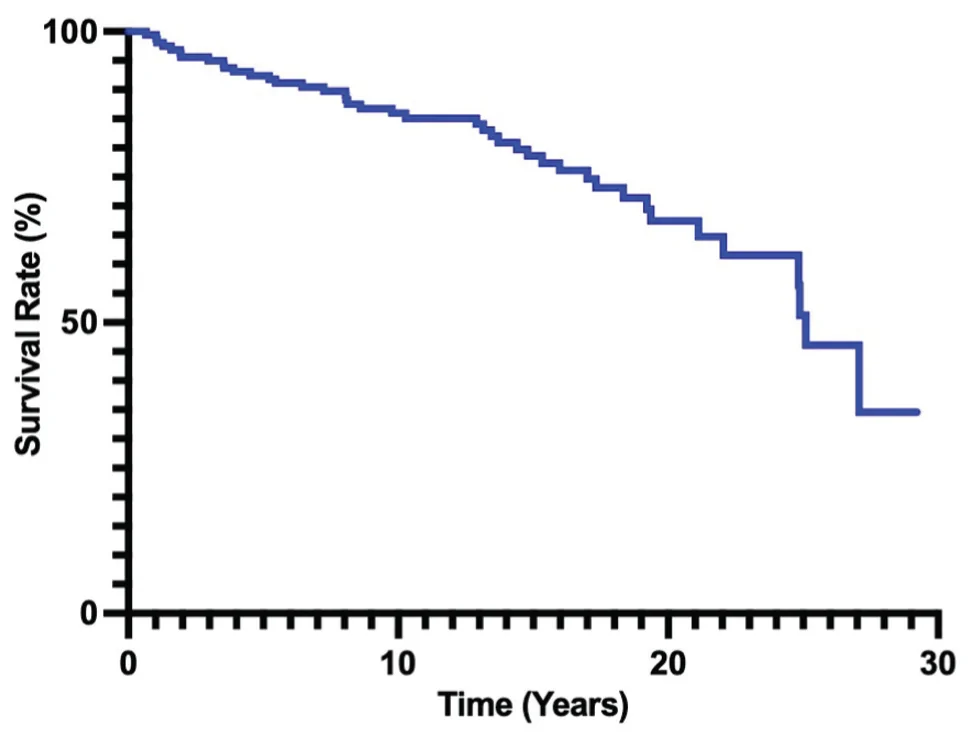
Overall cumulative meniscal allograft survival.

**Figure 3.**
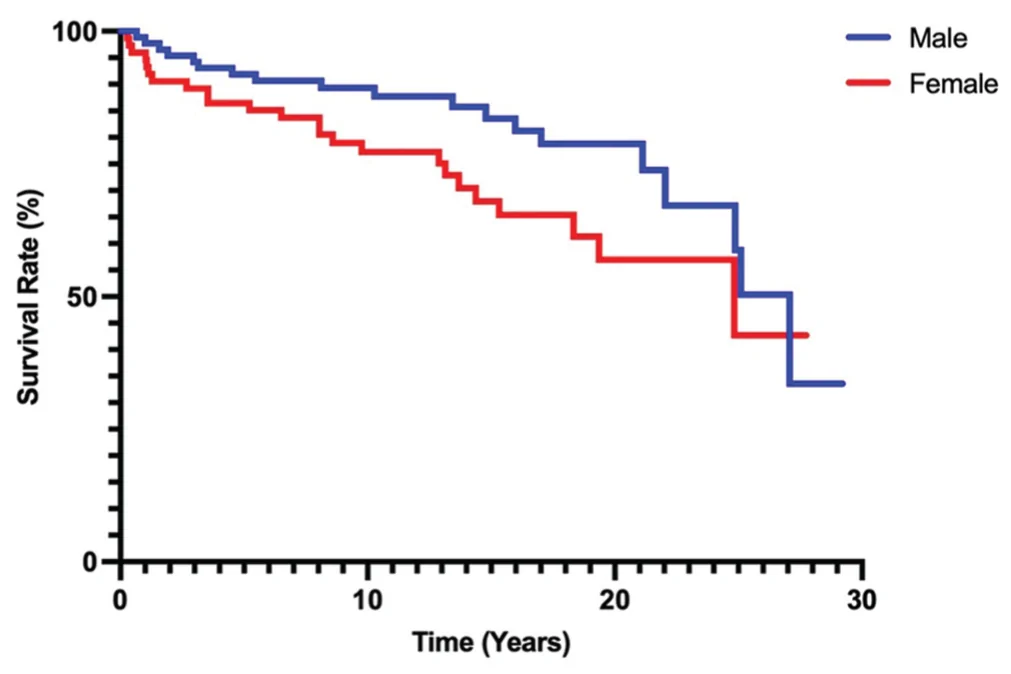
Comparison of Kaplan-Meier survival curves stratified by sex (log-rank *P* = .07).

A total of 43 graft failures occurred during follow-up, representing an 84% survival rate at 10 years, 76% at 15 years, 69% at 20 years, and 52% at 25 years (Figure 2). The median time to failure was 8 years (IQR, 2-15 years), with 17 patients (11%) experiencing graft failure within 5 years after MAT. Ten patients underwent osteotomy before or concomitantly with their MAT procedure, 4 of whom experienced graft failure. No variables, including fixation method (bone block vs all-suture), were significant risk factors for graft failure on univariable Cox regression (all *P* > .05) (Table 3). However, after adjusting for age and cartilage grade in the MAT compartment, female sex became a significant predictor on multivariable Cox regression analysis (*P* = .04) (Table 4). Survival patterns were compared between male and female patients, which were not significantly different (*P* = .07) (Figure 3).

**Table 3:** Univariable Cox Regression of Risk Factors for Graft Failure*^*a*^*

	Hazard Ratio	95% CI	*P* Value
Descriptive characteristics			
Age at surgery	1.0	1.0-1.1	.20
Female sex	1.8	1.0-3.2	.07
BMI at surgery	1.0	1.0-1.1	.55
Any tobacco use	1.8	0.9-3.3	.08
Follow-up length	1.0	0.9-1.0	.41
Surgical characteristics			
Lateral meniscus	1.3	0.7-2.4	.40
Bone block fixation technique	0.9	0.5-1.7	.77
ICRS grade ≥3 present in any compartment	1.2	0.7-2.2	.56
ICRS grade ≥3 present in MAT compartment	1.5	0.8-2.8	.18
No. of surgeries before MAT	1.0	0.9-1.2	.81
Time from first operative procedure of index knee	1.0	0.9-1.1	.84
Prior or concomitant osteotomy	1.2	0.5-2.6	.69

a BMI, body mass index; ICRS, International Cartilage Regeneration & Joint Preservation Society; MAT, meniscal allograft transplantation.

**Table 4:** Multivariable Cox Regression for Graft Failure*^*a*^*

	Hazard Ratio	95% CI	*P* Value
Age at surgery	1.0	1.0-1.1	.23
Female sex	1.9	1.0-3.6	**.04**
ICRS grade ≥3 present in MAT compartment	1.6	0.9-2.9	.15

a Variables were selected for inclusion in the Cox regression model based on clinical relevance and statistical significance in univariable analyses. Boldface type indicates statistical significance. ICRS, International Cartilage Regeneration & Joint Preservation Society; MAT, meniscal allograft transplantation.

### PROs and Clinical Survival Analysis

**Figure 4.**
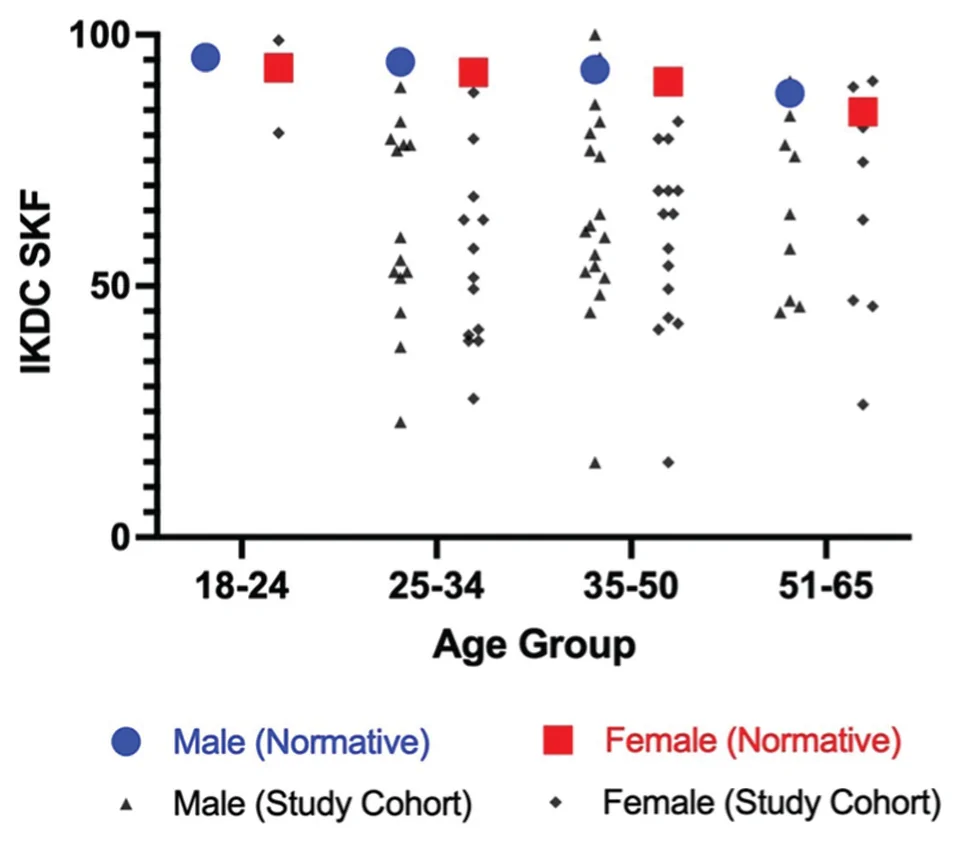
International Knee Documentation Committee Subjective Knee Form (IKDC SKF) scores of study cohort in relation to age- and sex-matched normative values.

PROs at the final follow-up appointment were available for 81 patients (51%). Of these, clinical failures (IKDC score <56) occurred in 32 patients (40%). Clinical failures were not associated with graft failures (*P* = .20). At the final follow-up, the mean IKDC SKF score of the overall cohort was 63 ± 20, and the mean Marx Activity Scale score was 4 ± 5. The mean standardized IKDC SKF score for the overall cohort was −2.6 ± 1.9, corresponding to a mean of 2.6 standard deviations below age- and sex-matched population normative values (Figure 4). A sex-based comparison of PROs among those with surviving grafts was not statistically significant (all *P* > .05) (Table 5). The same set of variables in Table 3 were evaluated for risk factors of clinical failure, and none were found significant on univariable logistic regression analysis (all *P* > .05).

**Table 5:** Comparison of Outcomes Between Male and Female Patients*^*a*^*

	Male (n = 86)	Female (n = 74)	*P* Value
Survivorship, % (events/total)			
Graft survival rate*^*b*^*	78 (67/86)	68 (50/74)	.14
Clinical survival rate*^*c*^*	63 (27/43)	59 (23/39)	.72
Surgical outcomes, n (%)			
Any reoperation on index meniscus	24 (28)	20 (27)	.90
Meniscal allograft repair	3 (3)	6 (8)	.21
Partial meniscectomy of meniscal allograft	14 (16)	5 (7)	.06
Total meniscectomy of meniscal allograft	5 (6)	6 (8)	.57
Secondary (revision) MAT	3 (3)	3 (4)	>.99
PROs in those with surviving grafts (n = 65)			
IKDC SKF	67 ± 19	60 ± 19	.09
Standardized IKDC SKF score*^*d*^*	−2.7 ± 2.2	−2.7 ± 1.7	.97
KOOS			
Symptoms	72 ± 18	63 ± 18	.06
Pain	81 (67-94)	81 (65-86)	.39
ADL	90 (79-99)	90 (74-97)	.67
Sports and Recreation	58 ± 26	53 ± 27	.43
QOL	55 ± 26	51 ± 24	.54
Marx Activity Scale score	2 (0-4)	00 (0-6)	.76

a Parametric variables are reported as mean ± SD, while nonparametric variables are reported as median (IQR). ADL, Activities of Daily Living; IKDC SKF, International Knee Documentation Committee Subjective Knee Form; KOOS, Knee injury and Osteoarthritis Outcome Score; MAT, meniscal allograft transplantation; PRO, patient-reported outcome; QOL, Quality of Life.

b Graft failure is defined as meniscal allograft repair, removal of most or all of graft, or conversion to total knee arthroplasty.

c Clinical failure is defined as an IKDC score <56 (Patient Acceptable Symptom State cutoff for MAT as defined in prior studies) at the final follow-up.

d Age- and sex-adjusted deviation from population norms.

Return-to-sport data were available in 61 patients who indicated that they participated in a sport before their meniscal injury, with sport types including football, soccer, basketball, skiing, snowboarding, dance, cheer, and softball. Of these 61 patients, 24 (39%) returned to their primary sport after MAT. Regarding level of participation, 10 patients (42%) participated at the varsity level before their meniscal injury, and after MAT, this number fell to 5 patients (21%). Regarding frequency of participation, 13 patients (54%) participated 4 to 7 times per week before their injury. After MAT, 8 patients (33%) participated at this frequency.

## Discussion

The primary finding of this study was that MAT graft survival rates were 89% at 5 years, with a gradual decline to 84% at 10 years, 76% at 15 years, 69% at 20 years, and 52% at 25 years. Female sex was a significant predictor of graft failure when accounting for age and presence of a high-grade cartilage lesion in the MAT compartment. Long-term PROs after MAT were lower than expected for patients’ age and sex, and return-to-sport rates remained low.

The graft survivorship rates of the present study were modestly higher than those in the current literature. A recent 2026 systematic review of 13 studies and 632 MATs demonstrated survivorship of ≥73% at a minimum 10-year follow-up, with survival after 15 years ranging from 19% to 87%.^16^ A 2019 systematic review of 11 studies and 688 MATs with a minimum 10-year postoperative follow-up reported a mean graft survivorship rate of 73.5% at 10 year and 60.3% at 15 years.^23^ A meta-analysis of 9 studies and 694 MATs with a minimum 5-year follow-up reported that 85.8% of medial and 89.2% of lateral MAT grafts survived in the midterm (5-10 years), while 52.6% of medial and 56.6% of lateral MAT grafts survived in the long term (>10 years).^2^ However, studies included in these reviews utilized varying definitions for graft failure, ranging from partial graft removal, any reoperation related to the transplant, complete resection of graft, and conversion to TKA, to clinical criteria such as a Lysholm score <65 or the need for additional surgery. This makes direct comparisons between studies difficult. The present study used a previously established definition^38^ that most accurately reflects whether subsequent surgery reflects failure of the meniscal allograft's integrity and function.

In this study, female sex was a significant predictor of graft failure after accounting for age and compartment-specific cartilage quality, highlighting the interactions of factors relating to the patient as well as the joint. In a recent systematic review of 21 studies and 2533 patients, among preoperative risk factors that were frequently studied, high-grade cartilage defects were identified as the only factor consistently predictive of graft failure.^37^ However, many of these studies did not treat all cartilage lesions at the time of MAT. To minimize this source of bias, the current study excluded patients with untreated high-grade cartilage lesions. The current study is also unique in that cartilage lesions in the knee overall as well as in the specific compartment of MAT were evaluated separately for their effects on graft and clinical failures. Although the presence of a high-grade cartilage lesion was not an independent predictor of graft failure in this study, the presence of a grade 3 cartilage lesion in the MAT compartment specifically and age at surgery modified the effect of sex, such that female sex became a significant predictor. These observations parallel prior retrospective data identifying high-grade preoperative osteoarthritis of the index compartment as a major predictor of MAT failure.^39^ Sex-based differences have also been reported, with lower midterm graft survival and higher reoperation rates observed in females.^6,10,11^ It is possible that the higher failure rate in females reflects factors such as sex mismatch between donor grafts and recipients, differences in lower limb alignment, and greater baseline ligamentous laxity compared with males.^3,9,18,22,27^ While inherent sex-related differences in bony morphology such as dimensions of the distal femur, proximal tibia, and patella may account for the difference in graft survival or reoperation rates between males and females, it is possible that a complex interaction between sex, age, and cartilage quality collectively influences the risk of graft failure.^25^ As such, further studies comparing patterns of graft survival after MAT between male and female patients are warranted. It would also be valuable to further examine donor-recipient sex matching, given conflicting research on whether females receiving grafts from male donors have a higher failure rate.^18,22^

While graft survivorship is a key outcome after MAT, PROs are equally important to consider, given that MAT is increasingly regarded as a procedure that delays rather than prevents arthroplasty.^32^ Several studies have demonstrated significant long-term improvements in PROs such as the Lysholm score and KOOS.^11,19,26,28,35^ Although the cohort of this study demonstrated IKDC SKF scores lower than their age- and sex-matched normative values, it is important to consider the context and expectations surrounding persistent symptoms after meniscectomy. Given the lower scores, most patients may not consider their knee as fully “normal” at the final follow-up. However, many patients may still perceive these outcomes as satisfactory, given that they exceeded the PASS threshold for MAT^36^ and were able to avoid more invasive measures such as TKA at a relatively young age. Additionally, while female sex was identified as a risk factor for graft failure, PROs did not differ between sexes, aligning with previous reports.^6^ These findings suggest that although females may have a higher likelihood of graft-related complications, those who retain a surviving graft can achieve functional outcomes comparable to males.

This study has several limitations. First, an inherent limitation of this long-term study is that surgical techniques, devices, and surgeons’ experience evolved over time. Second, this study is limited by the heterogeneity of the cohort, including the high prevalence of concomitant procedures as well as staged and unplanned interventions before and after MAT. This may confound the interpretation of outcomes attributable to MAT alone. Finally, the study focused on graft survivorship, and PROs were not available for the entire cohort, which could introduce bias.

## Conclusion

In this study, MAT survival rate, defined as the absence of subsequent subtotal or total meniscal allograft resection or repair, revision MAT, or conversion to TKA, was 84% at 10 years, with a gradual decline to 76% at 15 years, 69% at 20 years, and 52% at 25 years. After adjusting for age and presence of a high-grade cartilage lesion, female sex emerged as a predictor of graft failure. These findings highlight the importance of careful patient selection and counseling regarding long-term expectations after MAT.
